# Biological Interactions in Rat Tibial Osteogenesis: Micro-CT, Histomorphometric, and Histological Analyses of Bioglass, Fibrin Biopolymer, and Photobiomodulation

**DOI:** 10.3390/biom16060783

**Published:** 2026-05-26

**Authors:** Lívia Maluf Menegazzo Bueno, Livia Cristina Dias, Ana Carolina Cestari Bighetti, Camila Pascoal Correia dos Santos, Benedito Barraviera, Rui Seabra Ferreira Júnior, Murilo Priori Alcalde, Marco Antonio Hungaro Duarte, Carlos Henrique Bertoni Reis, Daniela Vieira Buchaim, Rogerio Leone Buchaim

**Affiliations:** 1 Postgraduate Program in Applied Dental Sciences, Bauru School of Dentistry (FOB/USP), University of Sao Paulo, Bauru 17012-901, SP, Brazil; likamaluf@usp.br (L.M.M.B.); anacarolinacb@usp.br (A.C.C.B.); camila.pcs@usp.br (C.P.C.d.S.); 2 Dentistry School, University Center of Adamantina (FAI), Adamantina 17800-000, SP, Brazil; 3 Department of Biological Sciences, Bauru School of Dentistry (FOB/USP), University of Sao Paulo, Bauru 17012-901, SP, Brazil; liviacdias@usp.br; 4 Center for the Study of Venoms and Venomous Animals (Cevap), São Paulo State University (Univ. Estadual Paulista, UNESP), Botucatu 18610-307, SP, Brazil; benedito.barraviera@unesp.br (B.B.); rui.seabra@unesp.br (R.S.F.J.); 5 Medical School, São Paulo State University (Univ. Estadual Paulista, UNESP), Botucatu 18618-687, SP, Brazil; 6 Center for Translational Science & Biopharmaceuticals Development (CTS-Cevap), São Paulo State University (Univ. Estadual Paulista, UNESP), Botucatu 18610-307, SP, Brazil; 7 Department of Dentistry, Endodontics and Dental Materials, Bauru School of Dentistry (FOB/USP), University of São Paulo, Bauru 17012-901, SP, Brazil; malcalde@fob.usp.br (M.P.A.); mhungaro@fob.usp.br (M.A.H.D.); 8 UNIMAR Beneficent Hospital (HBU), University of Marília, Marília 17525-160, SP, Brazil; dr.carloshenriquereis@usp.br; 9 Medical School, University Center of Adamantina (FAI), Adamantina 17800-000, SP, Brazil; danibuchaim@alumni.usp.br; 10 Graduate Program in Anatomy of Domestic and Wild Animals, Faculty of Veterinary Medicine and Animal Science, University of São Paulo (FMVZ/USP), São Paulo 05508-270, SP, Brazil; 11 Department of Postgraduate, School of Dentistry, Faculty of Midwest Paulista (FACOP), Piratininga 17499-010, SP, Brazil

**Keywords:** bone regeneration, biomaterials, photobiomodulation, low-level laser therapy, fibrin sealant, fibrin biopolymer, bioglass, bioactive glass

## Abstract

The study evaluated bone repair in tibial defects of Wistar rats treated with 45S5 bioactive glass, either alone or combined with lyophilized heterologous fibrin biopolymer (HFB) and/or photobiomodulation therapy (PBM). Thirty-five animals were randomly assigned to five groups: control (CG), Bioactive glass (BG), Bioactive glass + HFB (BFG), Bioactive glass + PBM (BPG), and Bioactive glass + HFB + PBM (BFPG). After 42 days, the samples were analyzed by micro-computed tomography, histology (Hematoxylin–Eosin, Masson’s Trichrome, and Picrosirius Red), and histomorphometry. Histological and micro-CT findings demonstrated improved defect closure and better matrix organization in the BG and BFG groups. Histomorphometric analysis revealed significant differences among the groups (ANOVA, *p* < 0.0001), with the BG group showing the highest percentage of new bone formation (40.35 ± 4.14%), significantly higher than the BPG and BFPG groups. The addition of HFB did not impair bone repair and yielded intermediate results, whereas PBM did not demonstrate a positive effect on bone regeneration at the 42-day time point under the parameters used in this study. It can be concluded that bioactive glass, especially when used alone or in combination with heterologous fibrin biopolymer, promoted superior bone regeneration, while its association with photobiomodulation did not demonstrate additional benefit at 42 days.

## 1. Introduction

Bone tissue is a critical component of the human body, providing structural support and protection for both the skeleton as a whole and the oral apparatus in particular. Its principal cellular constituents include osteoblasts, which synthesize and deposit new bone matrix, and osteoclasts, which are specialized for the resorption of mineralized tissue [1]. The tightly regulated equilibrium between osteoblast-mediated bone formation and osteoclast-mediated bone resorption is essential for preserving skeletal integrity and maintaining bone homeostasis throughout life [2]. In dentistry, bone defects arising from tooth loss, congenital anomalies, systemic bone diseases, or local conditions that compromise the alveolar ridge can significantly complicate oral rehabilitation and strongly influence therapeutic planning [3,4]. In such scenarios, a staged approach involving targeted bone regeneration or augmentation prior to definitive rehabilitation is often required to optimize the prognosis and to support stable, functional, and esthetic treatment outcomes [5].

Bone tissue engineering has advanced through diverse grafting materials designed to restore native bone architecture with maximal fidelity [6]. Biomaterials, either naturally derived or synthetically engineered, must exhibit biocompatibility when interfacing with host tissues [7]. Particulate bone graft substitutes are widely employed to fill osseous defects and promote bone regeneration in periodontal and implant-related procedures, offering enhanced adaptability and minimally invasive alternatives to conventional autogenous graft harvesting [8].

Biomaterials have significantly advanced regenerative dentistry by enabling more predictable bone repair and reducing postoperative morbidity [9,10]. Among the available options, particulate graft materials such as hydroxyapatite, beta-tricalcium phosphate, xenografts, allografts, and synthetic bioceramics have demonstrated osteoconductive properties and the ability to support new bone formation [11,12,13,14,15].

The selection of biomaterials depends on factors such as defect morphology, surgical objectives, and biological characteristics of the recipient site [16]. These materials are available in different presentations, including blocks, granules, membranes, and microspheres, allowing their use in a wide range of clinical applications related to bone regeneration [17]. In general, these biomaterials exhibit mechanical characteristics compatible with mineralized tissues, particularly high compressive strength and low tensile strength [18].

Among bioceramics, glass-based materials stand out for their exceptional bioactivity and capacity to promote bone regeneration. Upon implantation, they react with surrounding body fluids, initiating a cascade of physicochemical and cellular events that culminate in the formation of a biologically derived hydroxyapatite layer on their surface, along with mineralized osteoid deposition [19]. This mechanism fosters strong integration with host bone, establishing a durable and cohesive interface. The bioactivity of bioglass, manifested through its capacity for bone bonding, controlled biodegradation, and apatite layer formation on its surface, is profoundly influenced by its precise chemical composition and the relative proportions of its constituent elements [20].

In osseous environments, bioglasses exhibit strong adherence to collagen, growth factors, and fibrin, thereby forming a porous matrix that enables efficient infiltration by osteogenic cells. While this scaffold provides moderate compressive resistance, it generally lacks the mechanical robustness required for substantial load-bearing support [21].

Bioglasses have gained prominence due to their excellent biocompatibility, osteoinductive and osteoconductive properties, high bioactivity, and, in certain formulations, their capacity to promote angiogenesis. Researchers have developed diverse compositions, from highly reactive variants that integrate with soft tissues to others optimized for superior mechanical strength or controlled biodegradation rates [19,20,22]. These materials find applications such as solid blocks, particulate granules, components within composites, or scaffolds that support cellular adhesion, proliferation, and differentiation. Incorporating bioglass into hydroxyapatite is designed to augment its performance by accelerating chemical dissolution and amplifying overall bioactivity [23,24].

Bone regeneration outcomes can be further optimized through adjuvant therapies. Indeed, research exploring combined therapeutic approaches has demonstrated accelerated healing rates and enhanced repair efficacy [25]. Fibrin sealants and photobiomodulation therapy stand out among adjuvant treatments for bone repair. Fibrin glue, a biocompatible surgical adhesive derived from blood plasma’s clotting protein, forms a provisional matrix that supports cell proliferation, neovascularization, and natural degradation during healing. Photobiomodulation, meanwhile, accelerates tissue repair while reducing contamination risks [26,27].

Researchers from the Center for the Study of Venoms and Venomous Animals (CEVAP) at São Paulo State University (UNESP, Botucatu, Brazil) have pioneered a snake venom-derived fibrin sealant that bypasses human blood components. Renowned as a heterologous fibrin biopolymer (HFB) for its versatile biological effects, this material has shown promising results in the regeneration of neural, dermal, and osseous tissues [28]. HFB was originally produced in 3 frozen vials, but currently a lyophilized version of the biopharmaceutical has been released for research purposes, which facilitates its storage, transport, and use.

Low-level laser therapy (LLLT), now termed photobiomodulation (PBM), serves as another valuable adjunct in bone repair. It accelerates tissue healing, mitigates inflammation, and enhances regeneration across diverse tissues by stimulating mitochondrial function, boosting ATP synthesis, and optimizing cellular metabolism. Low-level laser therapy can be delivered locally using red or infrared wavelengths, selected based on the target tissue depth to achieve optimal therapeutic effects in superficial or deeper structures. Previous experimental studies have also demonstrated that PBM may favor bone neoformation, angiogenesis, and osteoblastic activity, although its effectiveness depends on the irradiation parameters and treatment protocols employed [29].

Thus, this study aims to evaluate the bone repair process in tibial defects in rats, using a bioactive glass biomaterial, alone or in combination with lyophilized heterologous fibrin biopolymer, and low-level laser photobiomodulation therapy, which simultaneously emits red and infrared lasers.

## 2. Materials and Methods

The study was conducted in strict accordance with the Ethical Principles for Animal Experimentation established by the National Council for the Control of Animal Experimentation (CONCEA, Brazil). The project was approved under protocol no. 25003 by the Animal Use Ethics Committee (CEUA) of the Centro Universitário de Adamantina (FAI) on 11 February 2025.

Throughout the entire experimental protocol, continuous monitoring of the animals’ clinical and behavioral status was conducted to identify potential signs of pain or distress. Alterations in the usual behavioral pattern were assessed, including decreased activity, social withdrawal, irritability, or hyper-responsiveness. Additionally, possible changes in gait, body posture, and facial expression were evaluated. General health parameters, such as overall physical condition, food and water intake, and the occurrence of any intercurrent clinical signs, were also carefully monitored.

### 2.1. Animal Maintenance

The experimental design included 35 male *Rattus norvegicus* (Wistar strain), approximately 90 days old, with a mean body weight of 300 g. The animals were housed in appropriate animal facility installations under systematic environmental monitoring. They were kept in suitable cages, with four animals per cage, under a controlled 12 h light/dark cycle and a stable ambient temperature of approximately 21 °C, maintained through a climate control system and regularly verified by temperature measurements. Throughout the entire experimental period, the animals had free access to standard chow and water ad libitum, with no restrictions on locomotion.

The determination of the sample size (*n*) and the allocation of animals among the experimental groups were established based on widely recognized bioethical criteria in scientific research. In this context, the principles of the 3Rs (Replacement, Reduction, and Refinement) were adopted, guiding the rational use of animal models, the improvement of experimental techniques, and the responsible conduct of experimental research [30]. No priori statistical power calculation was performed, which is acknowledged as a limitation of the study.

### 2.2. Experimental Design

**Figure 1:**
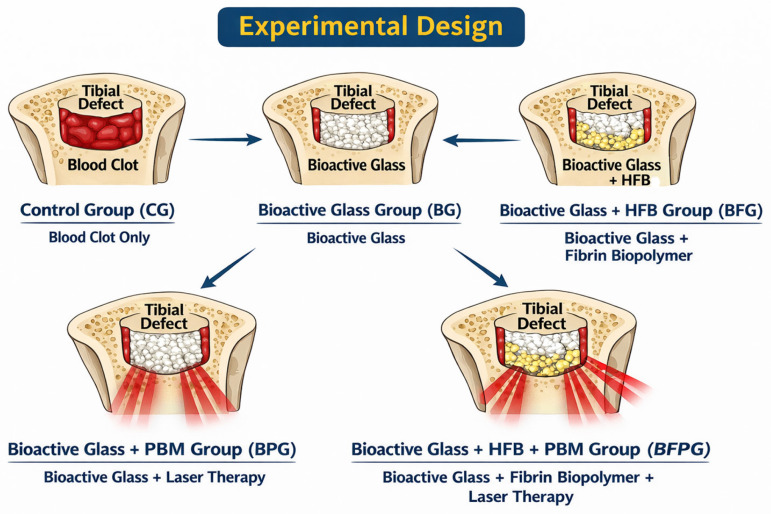
Animals were submitted to surgical creation of a tibial cavity defect and distributed into five groups: CG (blood clot only); BG (SinGlass^®^ bioactive glass; Síntegra Surgical, Pompéia, Brazil); BFG (bioactive glass + lyophilized HFB; CEVAP-UNESP, Botucatu, Brazil); BPG (bioactive glass + PBM using DMC Therapy EC^®^; São Carlos, Brazil); and BFPG (bioactive glass + lyophilized HFB + PBM).

The 35 animals were randomly allocated into five groups, with seven animals per group, distributed as follows (Figure 1).

#### 2.2.1. Lyophilized Heterologous Fibrin Biopolymer

Animals allocated to the BFG and BFPG groups received a previously standardized amount of lyophilized heterologous fibrin biopolymer, a material composed of the association of different biological components. This biomaterial consists of a serine protease (gyroxin) isolated from the venom of *Crotalus durissus terrificus* and a fibrinogen-rich cryoprecipitate obtained from the blood of *Bubalus bubalis* [31].

The biopolymer used in this study was supplied by the Center for the Study of Venoms and Venomous Animals (CEVAP, São Paulo State University—UNESP, Botucatu, Brazil) and provided in three separate vials: one containing fraction 1 composed of the lyophilized serine protease enzyme (gyroxin), another containing fraction 2 composed of lyophilized fibrinogen-rich cryoprecipitate, and a third vial containing the diluent, consisting of 10 mL of sterile water for injection. Biological active component 1, when combined with active component 2, forms a stable fibrin clot, which is subsequently diluted in sterile water for injection prior to application [28].

The product is protected by a patent registered under number BR 102014011432-7 (6 July 2022) [32]. The use of the biopolymer in its lyophilized form is justified by the limitations associated with the transport and handling of its liquid presentation, which is stored in vials that must remain frozen until the time of use. This requirement imposes logistical constraints and increases the risk of inadvertent thawing during storage or transportation, potentially compromising the stability and properties of the material [31,33].

When subjected to the lyophilization process, the biopolymer is converted into a powder form, which allows for greater ease and precision in handling, while significantly reducing the risk of physicochemical alterations resulting from temperature variations. Thus, the lyophilized form provides enhanced stability, safety, and practicality for both experimental and clinical management of the material [34].

The preparation of the heterologous fibrin biopolymer (HFB) followed the protocol described by the manufacturer. For this study, the following volumes were used: 20 μL of fraction 1, 20 μL of fraction 2, and 10 μL of diluent. The fractions were measured using Gibson^®^ micropipettes and disposable tips (Gilson Inc.^®^, Middleton, WI, USA).

#### 2.2.2. Bioactive Glass

The bioactive glass used in this study was SinGlass^®^ (Sintegra Surgical, Pompeia, Brazil), with according to the ASTM F1538-24 standard [35], which establishes criteria for the characterization and biocompatibility of bioactive glasses, this material used in this experimental protocol, has a specific proportion of its constituents: 45% silicon dioxide (SiO_2_), 24.5% sodium oxide (Na_2_O), 24.5% calcium oxide (CaO), and 6% phosphorus pentoxide (P_2_O_5_). This biomaterial exhibits angiogenic activity and antibacterial properties, characteristics that, together with its high bioactivity, reinforce its potential for biomedical applications [24,36].

#### 2.2.3. Photobiomodulation Therapy with Red and Infrared Laser

Animals in the BPG and BFPG groups underwent low-level laser therapy using the Therapy EC device (DMC^®^, São Carlos, Brazil), based on Gallium-Aluminum-Arsenide (GaAlAs). The protocol employed simultaneous emission in the red and infrared spectra, corresponding to wavelengths of 660 nm (Indium Gallium Aluminum Phosphide—InGaAlP) and 808 nm (Gallium Aluminum Arsenide—GaAlAs), respectively.

Application was performed in a punctual manner, directly at the center of the surgical lesion, with gentle contact with the tissue, for 30 s intraoperatively and subsequently three times per week until euthanasia. In all sessions, the equipment was properly calibrated and tested beforehand to ensure the accuracy of the administered dose.

**Figure 2:**
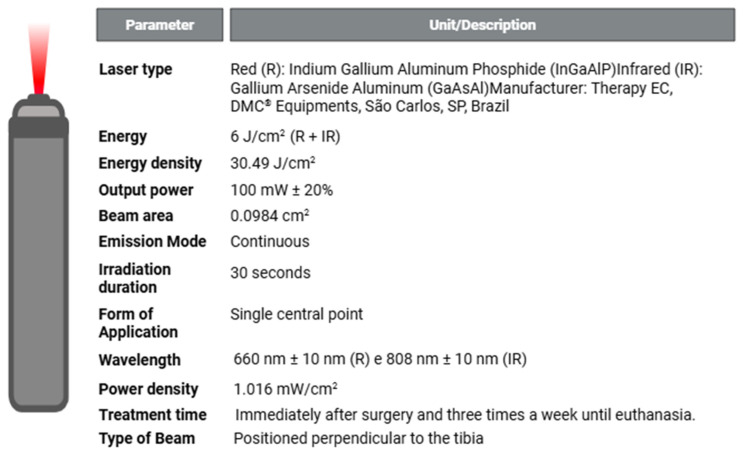
Details of the parameters used for the application of photobiomodulation therapy with the simultaneous emission of red and infrared spectra.

The physical parameters adopted included an output power of 100 mW (±20%), a beam area of approximately 0.098 cm^2^, and a total energy of 6 J (resulting from the combined application of the red and infrared wavelengths). Considering a power of 0.1 W and an exposure time of 30 s, the energy delivered per point was calculated using the equation E = P × t, totaling 6 J. Irradiance was determined by the ratio between power and beam area (P/A), reaching approximately 1.016 W/cm^2^. Fluence (energy density), obtained from the relationship between energy and the irradiated area (E/A), was approximately 30.49 J/cm^2^ when considering the total applied energy [37,38] (Figure 2).

### 2.3. Experimental Surgery

**Figure 3:**
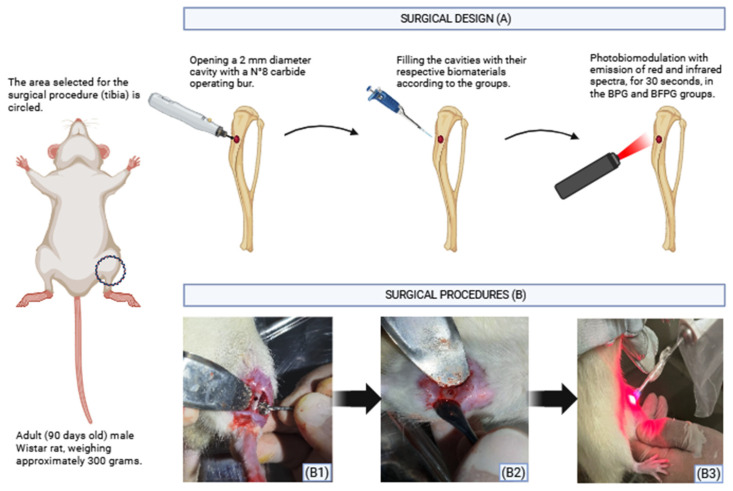
(**A**) Surgical design and representative intraoperative images. (**B1**) Creation of the tibial defect using a spherical Carbide bur under saline irrigation. (**B2**) Defect filled according to the experimental groups: CG, BG, BFG, BPG, and BFPG. (**B3**) Application of PBM with simultaneous red and infrared emission directly over the lesion site.

All surgical procedures were performed at the Bioterium of the Adamantina University Center (FAI, Adamantina, Brazil) by the same research team. For the experimental surgery, the rats were subjected to general anesthesia by intramuscular injection of ketamine (0.3 mL/kg; Dopalen^®^, Ceva Saúde Animal, Paulínia, Brazil) and xylazine (0.3 mL/kg; Anasedan^®^, Ceva Saúde Animal). After disinfection of the surgical field with 10% povidone-iodine topical solution, trichotomy was performed in the left hind limb. A linear incision of approximately 20 mm was then made in the craniocaudal direction, sectioning the skin and muscle fascia down to the periosteum, allowing exposure of the tibia. A 2 mm diameter cavity was prepared in the tibia using a spherical carbide tungsten surgical bur N° 8, coupled to a low-speed micromotor (1500 rpm), under constant irrigation with 0.9% saline solution (Figure 3).

All surgical procedures were performed individually on a protected surgical bench, with the table covered in cork and instruments replaced between animals. Thirty minutes prior to surgery, each animal received preemptive analgesia with tramadol hydrochloride (Tramal^®^, Grünenthal, São Paulo, Brazil) at a dose of 17.8 mg/kg and meloxicam (Meloxican^®^ EMS, Hortolândia, Brazil) at a dose of 1.5 mg/kg, both administered subcutaneously. The animals were positioned in ventral recumbency on the operating table, followed by local infiltration with 2% lidocaine with vasoconstrictor (Xylestesin^®^ Cristália, Itapira, Brazil) at a volume of 0.2 mL at the incision site, in order to improve hemostasis and local pain control.

Animals in the Control Group (CG) underwent only the surgical creation of the bone defect, allowing spontaneous blood clot formation within the cavity. For the groups treated with bioactive glass, approximately 0.04 g of the biomaterial was applied, previously measured using a precision balance to ensure dose standardization. For the groups that received the lyophilized heterologous fibrin biopolymer, the material was prepared and delivered using automatic micropipettes (Gilson Incorporated^®^, Middleton, WI, USA), ensuring accuracy and reproducibility of administration. The groups subjected to photobiomodulation therapy received low-level laser irradiation based on Gallium Aluminum Arsenide (GaAlAs), using the Therapy EC device (DMC Equipments^®^, São Carlos, Brazil). Irradiation was performed simultaneously at red wavelengths, via Indium Gallium Aluminum Phosphide (InGaAlP), and infrared wavelengths, via Gallium Arsenide Aluminum (GaAsAl), as described in Figure 2.

After cavity filling, the tissues in the operated region were repositioned and sutured using 4-0 silk sutures. All surgical procedures were performed by a single operator, ensuring that all animals were subjected to the same conditions.

Immediately after surgery, the animals received Tramadol at a dose of 17.8 mg/kg subcutaneously every 12 h for 3 days, and Meloxicam at a dose of 1.5 mg/kg subcutaneously every 12 h for 3 days.

After completion of this 3-day analgesic protocol, all animals received Dipirona^®^ (Novalgina Sanofi^®^, Suzano, Brazil—500 mg/mL) administered in a 400 mL drinking bottle, prepared with 4 mL of dipyrone and 396 mL of water, for a period of 5 days (Figure 3).

### 2.4. Surgical Procedure for Sample Collection

After a postoperative period of 42 days, seven animals from each group were euthanized, and the left hind limb was removed and the tibia dissected. The entire euthanasia procedure was carried out in a quiet environment, away from the other animals. A barbiturate (thiopental) was administered at a dose of 150 mg/kg for rats. Sodium thiopental 2.5% was delivered via intraperitoneal (IP) injection into the lower left abdominal quadrant of the animal, associated with local anesthetic (lidocaine hydrochloride at a dose of 10 mg/kg). After confirmation of death, the animal was placed in a white biological waste bag and sent for appropriate disposal.

### 2.5. X-Ray Micro-Computed Tomography Analysis

After fixation of the bone specimens (10% formaldehyde), morphometric analysis was performed using micro-computed tomography (micro-CT) with a SkyScan 1174v2 system (Bruker-microCT, Kontich, Belgium) at the Bauru School of Dentistry (Department of Endodontics, University of São Paulo, Bauru, Brazil). The device operated with an X-ray source set at 50 kV and 800 µA, a rotation step of 0.2°, a total rotation of 360°, and an isotropic resolution of 19.6 µm, resulting in an image acquisition time of approximately 35 min per sample.

The images obtained from each specimen were processed and reconstructed in both two- and three-dimensional formats, in the sagittal and transverse planes, to enable a detailed assessment of the structural characteristics of the bone tissue within the surgically created defect area. Image reconstruction was performed using NRecon software (version 1.6.9, Bruker-microCT), generating approximately 1000 to 1100 cross-sectional slices, according to the anatomical parameters established in the study.

The image assessment was performed qualitatively. Quantitative micro-CT analysis, including parameters such as bone volume fraction (BV/TV), trabecular thickness (Tb.Th), and trabecular number (Tb.N), was not conducted due to technical limitations in distinguishing newly formed bone from residual bioactive glass particles. The radiopacity of the biomaterial, which is comparable to or higher than that of mineralized bone tissue, may result in overestimation of bone-related parameters when automated segmentation methods are applied [32]. Furthermore, at the 42-day time point, a considerable amount of biomaterial remained within the defect area, further compromising the reliability of quantitative measurements. Therefore, qualitative analysis was considered more appropriate for evaluating defect closure, tissue organization, and biomaterial integration. To ensure accurate quantification of newly formed bone, complementary histomorphometric analysis was performed, which is regarded as a gold standard method for this purpose.

### 2.6. Sample Processing for Microscopy

After acquisition of the micro-computed tomography images, the samples were washed in running water for 24 h and then immersed in 10% EDTA (ethylenediamine tetraacetic acid), which was replaced every 7 days. During the EDTA replacement intervals, radiographic analyses were performed using Insight Kodak^®^ occlusal film (Eastman Kodak Company, Rochester, NY, USA) to confirm the decalcification process. After complete demineralization, the specimens underwent standardized histological processing.

Subsequently, 5 µm thick sections were obtained using a Leica RM2245 microtome (Leica Biosystems^®^, Wetzlar, Germany). Slides containing four to five sections were alternately stained with Hematoxylin–Eosin and Masson’s Trichrome for histomorphometric evaluation, and additional slides were stained with Picrosirius Red solution for collagen fiber assessment.

### 2.7. Histological Analysis

For the histomorphological description of the bone defect areas, the entire extent of each defect was considered in all specimens in order to evaluate the pattern of bone repair. This allowed the assessment, in each defect, of the presence of granulation tissue, inflammatory infiltrate, as well as the presence, quality (immature or mature/lamellar bone), and degree of filling of the newly formed tissue. Five semi-serial sections from the surgical bed of each defect were evaluated under a light microscope (Olympus BX50^®^, Olympus Corporation, Tokyo, Japan). Photomicrographs were captured using 4× and 10× objectives with an attached digital camera (Olympus DP71^®^, Olympus Corporration, Tokyo, Japan).

Histological and histomorphometric analyses were performed by a blinded examiner. The histological sections were previously coded to ensure that the evaluator was unaware of the experimental group allocation during analysis.

Quantitative image analysis, referring to the percentage of newly formed bone, was performed using a stereological point-counting method. The ImageJ software (version 1.50d, National Institutes of Health, Bethesda, MD, USA) was used as an auxiliary tool for image visualization and for superimposing a standardized grid over the histological images.

The estimation of newly formed bone was carried out using a grid composed of 88 equidistant points, following the principles described [39]. Points coinciding with newly formed bone tissue were counted and expressed as a percentage of the total number of points.

Histological sections were stained with Picrosirius Red solution and counterstained with Harris Hematoxylin. All sections were stained in the same batch to avoid any color variation among samples due to possible differences in dye impregnation. The birefringence intensity of collagen fibers promoted by Picrosirius Red staining was observed using a polarized lens coupled to an inverted Leica DM IRB/E microscope at the Integrated Research Center (CIPI/FOB/USP).

Images encompassing the entire histological section of the bone defect were captured using a 4× objective, which allows adequate visualization of repairing collagen fibers, including the thinnest fibers (exhibiting birefringence in yellow and red hues). Once an optimal light intensity was established on the optical microscope for fiber visualization, and the angle of the polarized lens was set at 90° relative to the microscope light source, all images were captured using the same parameters and saved in TIFF format at high resolution (1396 × 1626 pixels^2^).

Recent bone formation was quantified through morphometric analysis of histological images obtained at 4× magnification. For this procedure, a standardized point grid was superimposed over the area corresponding to the bone defect of each specimen. Points coinciding with newly formed bone tissue were counted and used to estimate the proportion of the area occupied by this tissue relative to the total analyzed area.

The grid consisted of 88 regularly distributed points, with total dimensions of 13.2 × 9.6 cm and uniform 1.2 cm spacing between points, mounted on a transparent support. The percentage of area occupied by newly formed bone was determined by the ratio between the number of points overlapping bone neoformation and the total number of grid points, multiplied by 100 (D = ΣPN/PT × 100). Results were expressed as the mean values obtained from all animals included in the analysis [39,40].

### 2.8. Statistical Analysis

Data regarding the percentage of newly formed bone, obtained by histomorphometry, were analyzed using GraphPad Prism (version 8.0; GraphPad Software, La Jolla, CA, USA). Bartlett’s test was applied to verify homogeneity of variances (*p* > 0.05), followed by one-way ANOVA and Tukey’s post hoc test (significance level α = 0.05). Quantitative histomorphometric comparisons were performed only among the treated experimental groups. Data were assumed to follow a Gaussian distribution based on the experimental design and sample characteristics; however, normality was not formally assessed using a specific statistical test, which should be considered a limitation.

## 3. Results

### 3.1. Micro-CT Results

**Figure 4:**
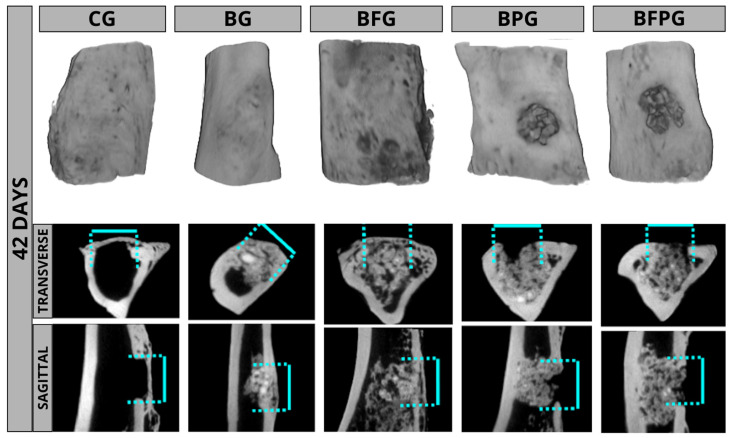
3D and 2D microtomographic images of defects over a period of 42 days. Composed of the following groups: CG (control group), BG (Bioactive glass), BFG (Bioactive glass + HFB), BPG (Bioactive glass + PBM), and BFPG (Bioactive glass + HFB + PBM). Blue dotted lines: a surgical defect created experimentally.

Figure 4 presents three-dimensional reconstructions and two-dimensional sections (transverse and sagittal planes) of rat tibiae at 42 days, allowing qualitative assessment of defect closure, organization of the newly formed tissue, and integration with the employed biomaterials.

In the control group, the bone defect persisted, leaving a large unfilled area. Three-dimensional images revealed a lack of cortical continuity, while two-dimensional sections showed an extensive central cavity with irregular borders and minimal deposition of mineralized tissue. These findings indicate a limited repair process, consistent with the low intrinsic capacity for spontaneous regeneration in critical-sized defects.

In contrast, the BG group exhibited superior regenerative outcomes, characterized by a marked reduction in the defect. Three-dimensional reconstructions demonstrated a clear decrease in lesion size, with dense and homogeneous bone formation. In both transverse and sagittal sections, substantial filling of the defect area with mineralized tissue was observed, along with integration of particulate material into the newly formed bone. These results suggest a high osteoconductive potential of the bioactive glass, promoting bone tissue deposition and maturation.

Similarly, the BFG group also shows clear evidence of bone regeneration, comparable to that observed in the CG and BG groups. The 3D reconstruction reveals cortical closure of the defect, while the 2D sections display a more continuous bone matrix characterized by thicker trabeculae and improved structural organization. The fibrin biopolymer is not detectable, with only the bioactive glass particles remaining visible.

The BPG group qualitatively demonstrates an intermediate degree of regeneration. Although bone neoformation is evident at the defect margins and in areas of mineralization within the lesion, closure remains partial and less homogeneous compared with the BG and BFG groups. The 2D reconstruction reveals persistent non-mineralized spaces, indicating incomplete repair at 42 days.

Finally, the BFPG group exhibits discrete to moderate bone formation. Despite the presence of residual material within the defect, both 3D reconstructions and 2D sections reveal less organization of the newly formed tissue and irregular filling, failing to reach the same level of closure observed in the BG and BFG groups. Overall, 3D and 2D microtomographic analyses demonstrated more pronounced evidence of defect closure in the CG, BG, and BFG groups; however, in the control group, the newly formed cortical bone appeared significantly thinner than the original.

The BG and BFG groups displayed the best regenerative outcomes among the treated groups, with partial to complete bone formation and variable integration of the biomaterial. Conversely, the BPG and BFPG groups exhibited the poorest results, characterized by persistent open defects and extensive hypodense regions, indicating a lack of bone neoformation.

### 3.2. Histomorphological Results

Histological analysis was performed using three staining methods: Hematoxylin and Eosin (HE), which allows global visualization of cellular components and the bone tissue matrix; Masson’s Trichrome, used to highlight connective matrix and collagenous structures; and Picrosirius Red, applied to identify collagen fibers, particularly useful for distinguishing between mature and immature collagen under polarized light.

For standardized interpretation, the following visual markers were adopted: an orange asterisk for connective tissue, a yellow asterisk for newly formed bone, and a green asterisk for residual biomaterial.

Over the 42-day period, a distinct histological pattern was observed among the experimental groups. The BG (Bioactive glass group) and BFG (Bioactive glass + heterologous fibrin biopolymer group) exhibited the most expressive results, both in terms of tissue organization and regenerative performance.

In the HE staining, new bone formation was clearly evident in the defects of the groups treated with Bioactive glass alone or combined with the lyophilized heterologous fibrin biopolymer. Extensive areas of maturing bone tissue (indicated in yellow) were observed, surrounding and integrating with the implanted biomaterial (indicated in green).

In contrast, the BPG (Bioactive glass + PBM) and BFPG (Bioactive glass + heterologous fibrin biopolymer + PBM) groups exhibited a lower proportion of newly formed bone tissue, with a predominance of connective tissue (indicated in orange) interposed among the biomaterial fragments. This pattern suggests a less efficient regenerative process, as the presence of interposed connective tissue generally reflects a delay in ossification. The CG, characterized as the control group, displayed a histological pattern consistent with the physiological bone repair process, without the influence of biomaterials or additional interventions.

**Figure 5:**
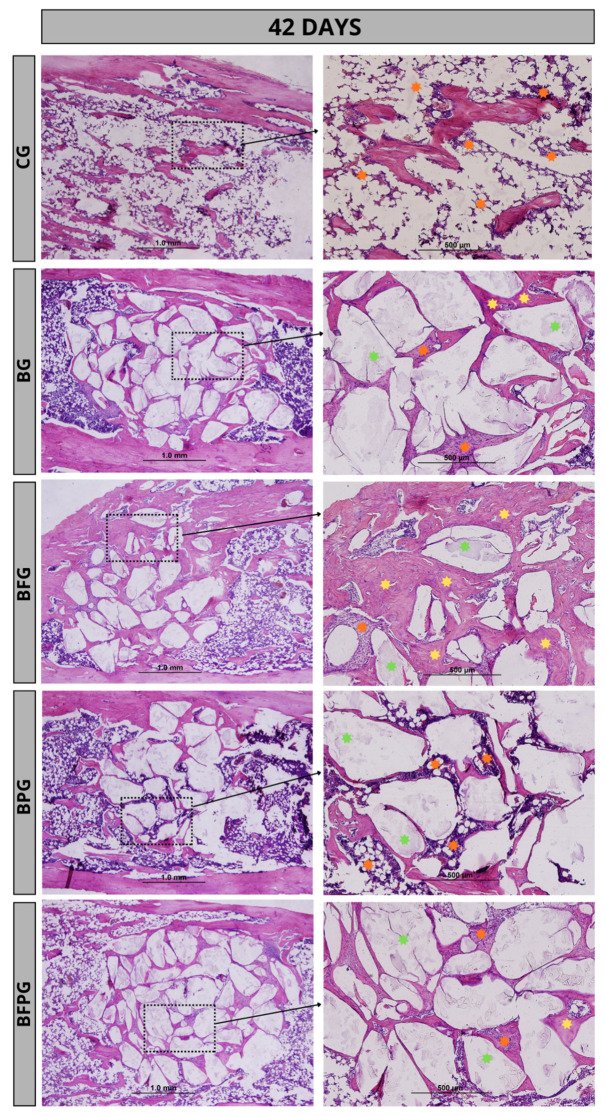
Histological sections stained with Hematoxylin and Eosin at 42 days for groups CG, BG, BFG, BPG, and BFPG at 4× and 10× magnifications. Orange asterisks indicate connective tissue, yellow asterisks newly formed bone, and green asterisks residual biomaterial.

Compared to the other experimental groups, the CG demonstrated more limited bone regeneration, predominantly characterized by the presence of fibrous connective tissue, discrete areas of new bone formation, and a greater extent of immature medullary spaces. This finding was expected, since in the absence of osteoconductive or osteoinductive biomaterials, repair occurs spontaneously and depends exclusively on the host’s natural biological mechanisms (Figure 5).

Masson’s Trichrome staining reinforced these findings. In the BG and BFG groups, intense collagen staining and the presence of mature bone matrix were observed, evidenced by more intense and well-organized bluish areas.

**Figure 6:**
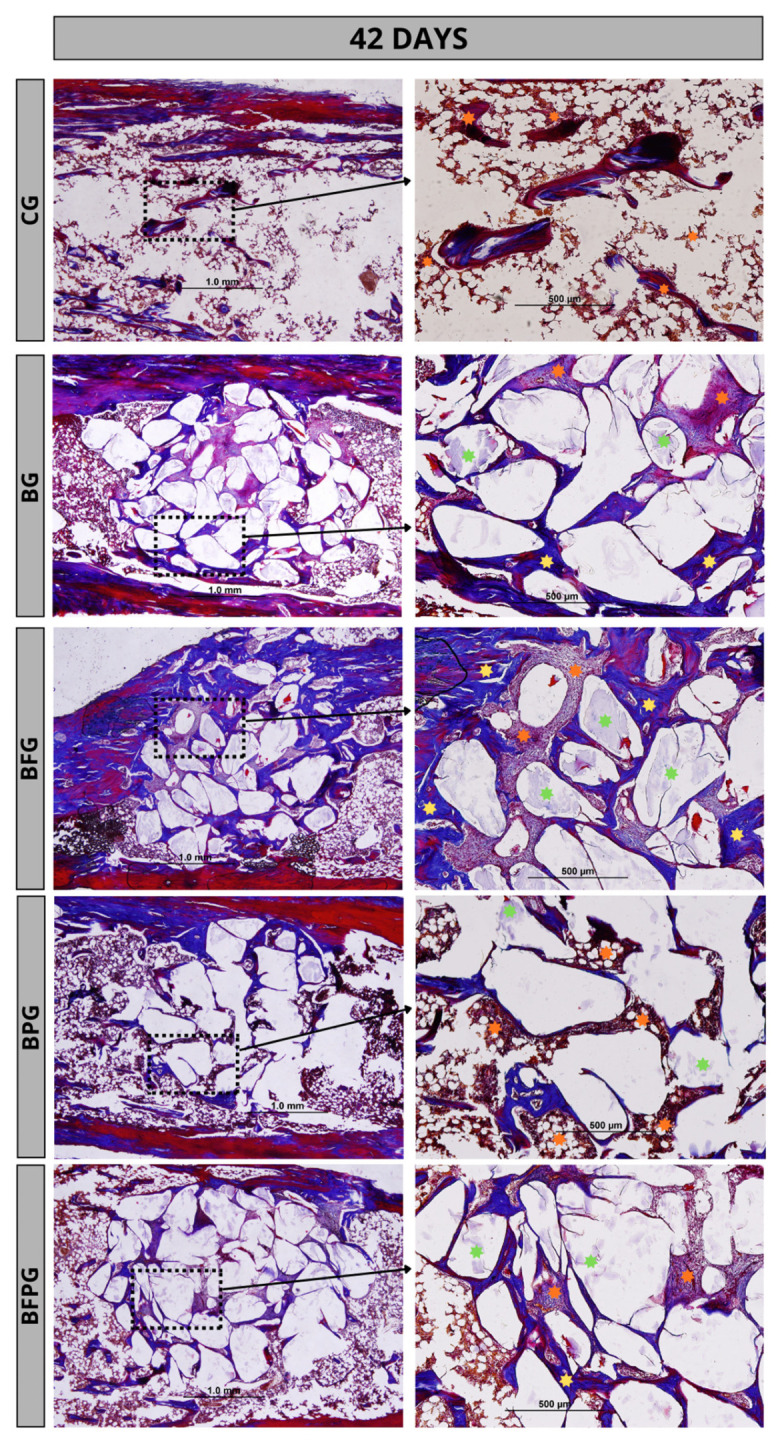
Histological sections stained with Masson’s Trichrome at 42 days for groups CG, BG, BFG, BPG, and BFPG at 4× and 10× magnifications. Orange asterisks indicate connective tissue, yellow asterisks newly formed bone, and green asterisks residual biomaterial.

In contrast, the BPG and BFPG groups, more markedly in the BPG group, showed a predominance of immature collagen fibers, less dense and weakly stained, accompanied by scarce bone matrix, indicating a delay in the remodeling and mineralization process (Figure 6).

Picrosirius Red staining revealed marked differences in the organization and maturation of collagen fibers among the experimental groups after 42 days. In the BG and BFG groups, there was a predominance of thick, highly organized collagen fibers exhibiting strong birefringence under polarized light, compatible with mature collagen (predominantly type I), indicating an advanced stage of bone remodeling. These findings corroborate the results observed with Hematoxylin and Eosin and Masson’s Trichrome staining, which had already demonstrated a greater amount of newly formed bone and a more organized bone matrix in these groups.

In contrast, the BPG and BFPG groups showed a higher proportion of thin, disorganized collagen fibers with reduced birefringence, characteristics of immature collagen (mainly type III), suggesting delayed extracellular matrix maturation and mineralization. This is consistent with the greater presence of connective tissue observed in the other staining methods.

**Figure 7:**
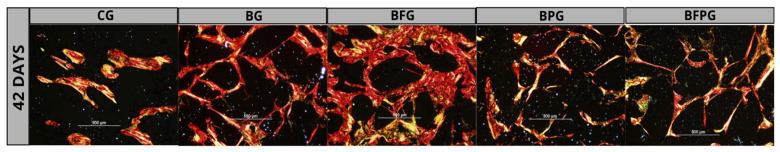
Histological sections stained with Picrosirius Red at 42 days. The figure comprises the groups CG (control group), BG (Bioactive glass), BFG (Bioactive glass + HFB), BPG (Bioactive glass + PBM), and BFPG (Bioactive glass + HFB + PBM), presented at a microscopic magnification of 4×.

The CG exhibited a pattern compatible with physiological bone repair, with a predominance of immature collagen and limited fiber organization, reflecting a slower and less efficient regenerative process compared to the treated groups (Figure 7).

Among all groups, BG and BFG exhibited the most favorable histological profile, combining a high amount of newly formed bone, advanced collagen fiber maturation, and minimal residual connective tissue. In contrast, the BPG group proved to be the least favorable, showing a predominance of immature collagen, low mineralization, and reduced bone formation.

**Figure 8:**
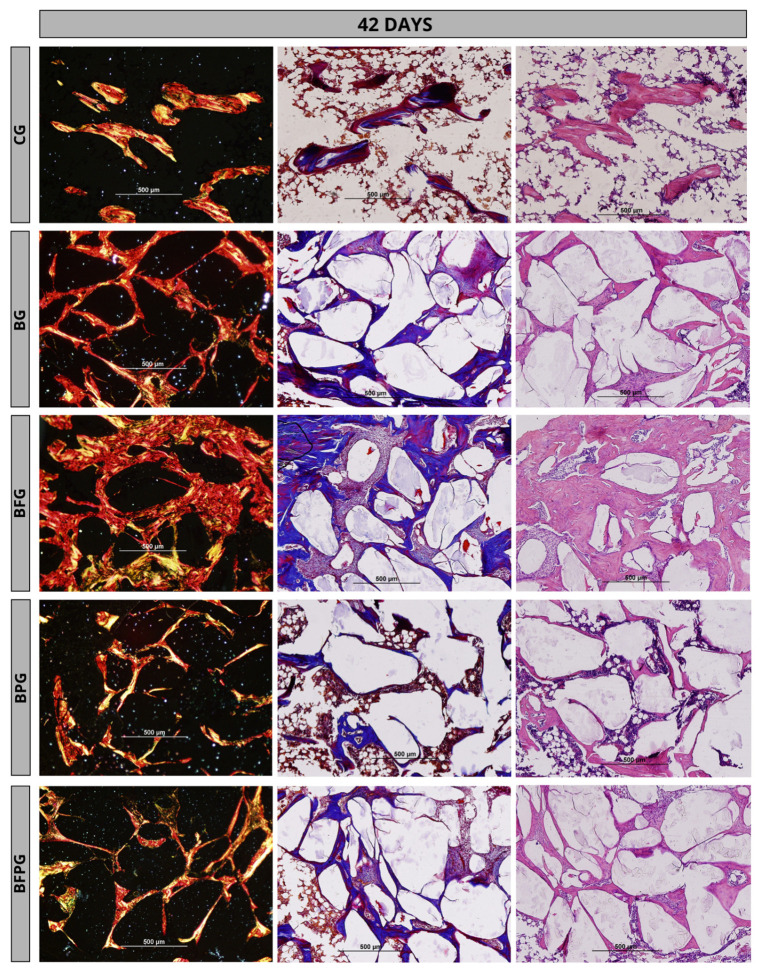
Histological sections stained with Picrosirius Red, Masson’s Trichrome, and Hematoxylin and Eosin, respectively, at 42 days. The figure comprises the groups CG (control group), BG (Bioactive glass), BFG (Bioactive glass + HFB), BPG (Bioactive glass s + PBM), and BFPG (Bioactive glass + HFB + PBM), presented at a microscopic magnification of 4×.

Overall, the findings obtained from the different staining methods demonstrate consistency and convergence toward the conclusion that the biomaterials used in the BG and BFG groups achieved superior performance in bone regeneration at 42 days. Conversely, the BPG group showed a significantly inferior response, indicating that the association of bioactive glass with photobiomodulation was not beneficial for the injured bone tissue (Figure 8).

In this context, the histological findings at 42 days demonstrate that the BG and BFG groups showed superior osteoregenerative performance, with a more organized matrix and a predominance of mature collagen. In contrast, the BPG and BFPG groups showed delayed repair, characterized by a higher presence of connective tissue and immature collagen, indicating that photobiomodulation did not result in improved bone regeneration at the 42-day time point under the parameters used in this study. The control group exhibited a pattern consistent with physiological repair; however, with lower regenerative efficiency compared to the treated groups.

### 3.3. Histomorphometric Results

The histomorphometric analysis performed at 42 postoperative days revealed significant differences among the groups regarding the percentage of new bone formation (ANOVA: F(3,24) = 12.48; *p* < 0.0001), indicating a treatment effect on the regenerative process (Table 1).

**Figure 9:**
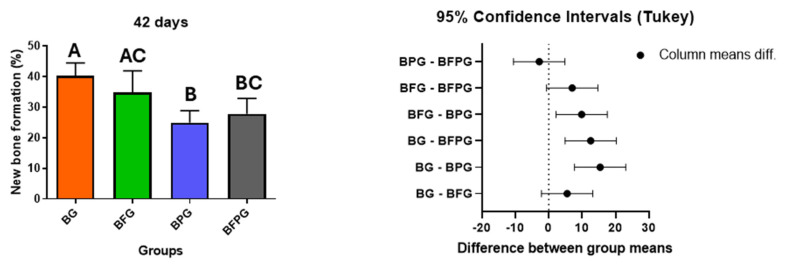
Histomorphometric analysis of new bone formation at 42 days. BG: bioactive glass; BFG: bioactive glass + HFB; BPG: bioactive glass + PBM; BFPG: bioactive glass + HFB + PBM. Different capital letters indicate statistically significant differences among groups.

The BG group exhibited the highest mean (40.35 ± 4.14), significantly higher than BPG (24.97 ± 3.87) and BFPG (27.80 ± 5.09), but not BFG (34.83 ± 7.08). The control group was not included in the quantitative statistical comparison due to the absence of relevant bone neoformation. BFG differed only from BPG, while BPG and BFPG did not differ from each other (Figure 9).

The BG group presented the highest percentage of newly formed bone, being statistically superior to the BPG and BFPG groups, as indicated by the different uppercase letters in the graph. The BFG group demonstrated intermediate values, not differing statistically from the BG group, but showing a significant difference compared to the BPG group.

The BPG and BFPG groups exhibited the lowest percentages of bone formation, with no statistically significant difference between them, characterizing similar biological performance whether or not photobiomodulation was associated in the presence of the biopolymer.

The 95% confidence interval graph (Tukey test) confirms these findings, demonstrating that the comparisons BG vs. BPG and BG vs. BFPG show positive mean differences with intervals that do not cross zero, evidencing statistical significance. The comparison between BFG and BPG also indicates a significant difference, whereas the remaining combinations present intervals that intersect zero, corroborating the absence of statistical difference among those groups.

Overall, the graphical results reinforce that the isolated use of bioactive glass (BG) promoted greater bone regeneration at 42 days, while its association with the biopolymer and/or photobiomodulation did not result in a significant additional increase when compared to the BG group, although the BFG group exhibited an intermediate response superior to the BPG group.

## 4. Discussion

This study aimed to comparatively evaluate bone neoformation in the tibia of rats through the use of bioactive glass (BG), applied either alone or in association with adjuvants, such as heterologous fibrin biopolymer and photobiomodulation with low-level laser therapy. The results demonstrate that the use of bioactive glass 45S5, whether associated or not with the heterologous fibrin biopolymer (HFB), shows favorable performance in bone neoformation, whereas its association with laser photobiomodulation did not demonstrate additional benefits for bone regeneration under the conditions evaluated in this study.

The use of the rodent tibia in this study is justified by the application of long bones in bone repair research, owing to both their ease of handling and access, and their ability to realistically reproduce biomechanical conditions found in humans. In these structures, the periosteum plays a fundamental role, functioning as support for bone regeneration studies and serving as a basis for tissue engineering investigations, especially in analyzing bone response to muscular loads and remodeling processes [41,42,43].

Developed by Larry Hench, BG has been used in the healthcare field since 1994, standing out for its ability to bind directly to bone tissue without the formation of a fibrous capsule, acting as a scaffold for bone regeneration. Bioactive glass promotes bone regeneration through its dissolution, with the release of biologically active ions capable of stimulating osteogenic cells, as demonstrated in in vitro studies [44,45]. Thus, this compound favors the adhesion, proliferation, differentiation, and mineralization of osteoblasts. In this light, the present study demonstrated that bioactive glass is capable of promoting bone neoformation, both in isolation and when associated with other therapies such as HFB, validating and reaffirming the findings reported in the literature [5,20,45,46,47].

Bioactive glass demonstrates a significant capacity to stimulate bone neoformation, as observed in this study, as well as a promising combination in bone regeneration when this compound was associated with HFB, as evidenced in BFG. The combination of the release of biologically active ions from bioactive glass with the stimulation of new vessel formation, three-dimensional structure, and growth factors promoted by fibrin compounds, such as HFB, results in a biocomplex with a microenvironment favorable to bone recomposition [24,34,44].

Snake venom-derived fibrin biopolymer has been extensively studied for its behavior as a biodegradable scaffold, capable of promoting cell adhesion, favoring tissue organization, and exhibiting both hemostatic and biodegradable properties. Furthermore, this material is capable of gradually releasing bioactive molecules at the implantation site. Due to these characteristics, HFB has been investigated as a bioactive pharmacological component, showing favorable results in applications related to tissue healing, nerve regeneration, and bone regeneration. Studies, such as those evaluating alveolar bone healing after tooth extraction, have demonstrated that the heterologous fibrin biopolymer was able to suppress osteoclastic activity, consequently reducing the bone resorption induced by the procedure. Thus, the use of this component for the control of clastic activity, associated with the stimulation of bone progenitors, may represent an important ally in dental practice [18,48,49,50,51].

Several studies involving HFB have demonstrated a favorable relationship between this compound and photobiomodulation, highlighting a reduction in the inflammatory process and stimulation of the formation of both the healing matrix and new bone tissue. However, in the present study, it was observed that the association of PBM with bioactive glass did not result in positive effects. As illustrated in Figure 8, the groups whose bone defects were filled with bioactive glass associated with PBM, with or without the presence of the fibrin biopolymer (BPG and BFPG), showed a predominance of immature collagen, low mineralization, and reduced bone formation [18,34].

Similar findings have been reported in studies evaluating the association between biomaterials and photobiomodulation therapy (PBM). Although both therapies individually demonstrate positive effects on bone repair, their combined use may not necessarily result in additive regenerative benefits [52,53,54]. In the present study, the absence of improved bone formation in the PBM-treated groups may be related to the irradiation parameters employed, since the biological effects of PBM are highly dependent on factors such as wavelength, energy density, irradiance, and application frequency [24,55,56].

The absence of additional regenerative effects associated with PBM in the present study may be related to the specific irradiation parameters employed. Previous studies have demonstrated that the biological effects of photobiomodulation are highly dependent on factors such as wavelength, energy density, exposure time, and treatment frequency. Variations in these parameters may lead to distinct cellular responses, including biomodulatory saturation or limited stimulation of osteogenic activity. In addition, the interaction between PBM and the biomaterial microenvironment may have influenced the observed outcomes.

PBM typically exhibits a biphasic dose–response behavior, in which lower fluences stimulate osteoblastic activity and tissue repair, whereas higher fluences may reduce these effects due to excessive reactive oxygen species production and altered cellular responses. The fluence applied in the present study (30.49 J/cm^2^) may have exceeded the optimal therapeutic window for this experimental model, which could explain the absence of additional regenerative effects. Similar outcomes have been reported in previous studies combining bioactive glass and low-level laser therapy, which also demonstrated limited or negative additive effects under higher irradiation parameters [53,57,58] (Table 2).

Additionally, studies using histological markers have shown that the association between bioactive glass and photobiomodulation may increase Runx-2 expression, indicating enhanced osteoblastic activity [60,61]. However, this increase does not necessarily result in greater bone neoformation, suggesting that the combined therapies may induce excessive or premature cellular stimulation during bone repair [52,62,63,64,65,66,67,68,69].

**Figure 10:**
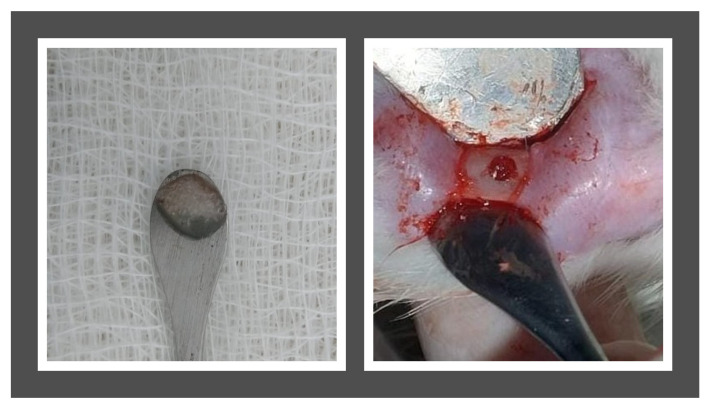
(**Left side**): Image of the association of lyophilized HFB, after careful reconstitution of its components, before surgical insertion, associated with bioactive glass (BG), constituting a biocomplex for filling the bone defect; (**Right side)**: Photographic image of the surgical cavity in the rat tibia, filled with HFB + BG, showing absence of bleeding and stability in the surgical bed.

In the present study, a novel lyophilized version of the heterologous fibrin biopolymer (HFB) was associated with the biomaterials. Unlike the conventional frozen formulation, the lyophilized HFB offers longer shelf life, easier storage and transport, and rapid reconstitution without requiring a cold chain, while maintaining its hemostatic and regenerative properties [70]. Furthermore, the material demonstrated adequate biodegradation, low immunogenicity, and the ability to form a stable fibrin network associated with the biomaterial, constituting the biocomplex previously described by our group [37] (Figure 10).

Although the findings of this study are promising, some limitations should be acknowledged. The 42-day evaluation period may not have been sufficient for complete integration and resorption of the analyzed biomaterials, as evidenced by the persistence of bioactive glass particles in the histological sections. Bone regeneration is a dynamic process, and the biological response observed at this time point may not fully reflect long-term outcomes. In this context, the relative performance of the experimental groups, particularly those subjected to photobiomodulation, may change at later stages of healing. In addition, the use of a single experimental model based on rats limits the direct extrapolation of these findings to human clinical settings. Furthermore, no a priori statistical power calculation was performed to determine the sample size, which represents an additional limitation. Although statistically significant differences were observed among the experimental groups, the possibility of insufficient statistical power cannot be excluded, particularly in comparisons that did not reach statistical significance, such as between the BG and BFG groups.

Another limitation of this study is the absence of a group treated exclusively with photobiomodulation, without the association of bioactive glass. Consequently, it is not possible to determine whether the lack of additional benefit observed in the PBM groups is specifically related to an interaction with the biomaterial or to the response of photobiomodulation itself under the experimental conditions adopted. Future studies including isolated PBM groups are necessary to better elucidate these mechanisms.

Although previous studies have reported limited additive effects between PBM and bioactive glass under certain irradiation conditions, the available literature remains heterogeneous regarding optimal irradiation parameters. Therefore, the present findings contribute to a better understanding of the biological limits and responses associated with this combined regenerative approach [71,72,73].

Therefore, further research involving larger sample sizes, different preclinical models, and longer follow-up periods is warranted to better understand the long-term performance of these materials. Future studies are also essential to elucidate the interactions between bioactive glass and other therapeutic protocols involving photobiomodulation.

Additionally, future studies should investigate different PBM irradiation parameters, longer follow-up periods, isolated PBM protocols, and comparisons between the present biocomplex and other bioinspired or naturally derived biomaterials, including marine-based scaffolds, in order to further understand their osteoconductive properties and interactions during bone repair.

## 5. Conclusions

In this preclinical study, bioactive glass SinGlass^®^ demonstrated favorable osteoregenerative performance in rat tibial defects, particularly when used alone or associated with the heterologous fibrin biopolymer, promoting greater bone neoformation and improved tissue organization. Conversely, the association of bioactive glass with photobiomodulation therapy did not enhance the regenerative process under the conditions evaluated in this study, particularly at the 42-day time point and with the parameters applied. Therefore, the combination of 45S5 bioglass and heterologous fibrin biopolymer appears to be a promising strategy for bone repair, while the interaction with PBM requires further investigation regarding its parameters and biological effects.

## Figures and Tables

**Table 1:** Comparison of mean percentage of new bone formed after 42 days of experimental surgery, between groups (BG, BFG, BPG, BFPG) by one-way ANOVA and Tukey. SD, standard deviation; 95% CI, confidence interval; Δ, difference of means; Ns, not significant (*p* > 0.05); ** *p* < 0.01; *** *p* < 0.001; **** *p* < 0.0001 (Tukey), ANOVA: F(3.24) = 12.48; *p* < 0.0001.

Group	N	Average ± SD	IC 95%	Tukey vs. BG	Tukey vs. BFG
BG	7	40.35 ± 4.14	36.52–44.19	-	Ns
BFG	7	34.83 ± 7.08	28.28–41.38	Ns	-
BPG	7	24.97 ± 3.87	21.38–28.55	**** (Δ = 15.38)	** (Δ = 9.866)
BFPG	7	27.80 ± 5.09	23.09–32.51	*** (Δ = 12.55)	Ns

**Table 2:** Comparison of PBM protocols with bioactive glass.

Protocol	Fluence (J/cm^2^)	Irradiance (W/cm^2^)	Outcome in Rat Tibia + BG	Reference
Present study	30.49	1.016	Poorer bone formation	-
Oliveira (2010)	120 total	Not specified	−17–31% new bone	[53]
Moreira (2018)	210	Not specified	No benefit (31% ANFB)	[58]
Ideal dose	4–16	0.02–0.3	Osteogenic stimulation	[59]

BG: Bioactive glass; ANFB: Areas of newly formed bone.

## Data Availability

The data presented in this study are available on request from the corresponding author.
